# Endemic and invisible: Visceral Leishmaniasis a One Health imperative in the Somali’s fragile health landscape

**DOI:** 10.1371/journal.pntd.0013905

**Published:** 2026-01-08

**Authors:** Hassan Sh. Abdirahman Elmi, Mohamoud Abdulahi, Abdirashid Abdirahman Hussein, Mohamoud Ahmed, Khadra Jama-Alol, Halima Mohamed

**Affiliations:** 1 School of Postgraduate Studies and Research, Amoud University, Borama, Somalia; 2 Department of Zoology, Faculty of Science, Charles University, Prague, Czech Republic; 3 School of Medicine, College of Health Science, Amoud University, Borama, Somalia; 4 Department of Pediatrics and Child Health, Jigjiga University Sh. Hassan Yabare Comprehensive Specialized Hospital, Jigjiga, Ethiopia; 5 Puntland State University, Garowe, Puntland, Somalia; 6 Buro University, Buro, Somaliland; 7 Nuffield Department of Primary Care Health Sciences, University of Oxford, Oxford, United Kingdom; University of Sao Paulo: Universidade de Sao Paulo, BRAZIL

## Abstract

Visceral Leishmaniasis (VL) remains one of the most neglected public-health challenges in the Horn of Africa. This editorial highlights the ecological, social, and political determinants driving VL persistence across the Somali Peninsula, including Somalia, the Somali Region of Ethiopia, and northeastern Kenya. It synthesises evidence on vector ecology, diagnostic and treatment gaps, and the humanitarian dimensions of disease vulnerability, arguing for a One Health, primary-care-anchored approach to prevention and control. The paper calls for renewed national commitment, regional coordination, and donor alignment to integrate VL into health-system-strengthening and universal health coverage agendas in fragile settings.

## Background

Visceral Leishmaniasis (VL), or kala-azar, remains one of the most severe yet neglected tropical diseases in the Horn of Africa. Its persistence across the Somali Peninsula embodies the intersection of fragility, poverty, and ecological vulnerability. Despite periodic outbreaks and high case fatality rates, VL continues to receive minimal attention in national policy and international humanitarian agendas. The disease is endemic across Somalia, the Somali Region of Ethiopia, and northeastern Kenya, where protracted conflict, climate variability, and population mobility have undermined control efforts for decades.

The ecological and social conditions that shape transmission in this region are unique. Pastoralism, seasonal migration, and frequent human–animal contact create ideal circumstances for the spread of *Leishmania donovani* by *Phlebotomus* sandflies [1,2]. Nomadic communities move across porous borders in search of pasture and water, often settling in temporary encampments where health services are absent and vector habitats proliferate. Recurrent droughts and displacement have exacerbated these patterns, forcing millions into unsanitary settlements that favour sandfly breeding [3]. Environmental changes linked to deforestation, unregulated urbanisation, and post-rainfall vegetation growth further sustain the vector population, while the absence of systematic vector surveillance precludes early warning and targeted intervention [4].

Health system fragility magnifies ecological risk. Most primary healthcare facilities in Somalia lack basic diagnostic capacity. The rK39 rapid diagnostic test and parasitological confirmation are rarely available outside major hospitals, and laboratory infrastructure remains limited [5]. Consequently, VL is frequently misdiagnosed as malaria or typhoid fever until patients present with advanced disease, by which time mortality approaches 90 percent in the absence of treatment [6]. The recommended therapy—liposomal amphotericin B—is either unavailable or prohibitively expensive, and alternative regimens carry high toxicity. Supply chains are erratic, subject to border closures and insecurity, leaving large areas without lifesaving drugs [7]. Humanitarian organisations fill some gaps, but coverage is patchy and episodic.

The humanitarian dimensions of VL are profound. Displacement, malnutrition, and conflict converge to drive transmission and mortality. Internally displaced persons and pastoralist families in informal settlements face overcrowding, poor housing, and limited access to clean water and nutrition—all factors that increase exposure and reduce immunity [8]. Malnutrition in particular impairs cell-mediated immune responses, heightening susceptibility and case fatality. Population mobility and conflict-related insecurity also disrupt case reporting and treatment adherence, masking the true burden of disease and compromising surveillance [9]. In such contexts, VL is not merely a medical condition but an indicator of systemic neglect and the collapse of social protection systems.

An effective response demands integration across human, animal, and environmental health sectors within a comprehensive One Health approach. Although humans are the principal reservoir of *L. donovani* in East Africa, livestock movements and environmental changes strongly influence vector ecology and transmission dynamics [10]. Collaboration between ministries of health, livestock, and environment is essential for mapping hotspots, monitoring vector densities, and implementing environmentally sensitive control strategies. Embedding VL activities within strengthened primary health care offers a realistic entry point for sustainability. Community health workers can be trained to recognise signs and symptoms, perform rapid tests, and initiate referrals. Integrating VL into existing malaria and cholera surveillance systems could maximise efficiency and build synergies for integrated disease control [5].

Somalia’s *One Health Strategy 2023–2035* creates a policy framework for multisectoral collaboration on zoonotic and vector-borne diseases [3]. To translate this vision into action, governments must invest in workforce training, interoperable data systems, and predictable financing for essential NTD commodities. Cross-border coordination with Ethiopia and Kenya is crucial to address mobile populations and harmonise surveillance standards. Operational research is urgently needed to characterise vector ecology, treatment outcomes, and behavioural determinants of care-seeking. Universities in the region—including Amoud University, the University of Jigjiga, and the University of Oxford—are well-placed to lead collaborative studies that generate context-specific evidence and strengthen local capacity.

The persistence of VL in the Somali Peninsula is a symptom of broader governance failures in fragile states where neglected diseases fall between the mandates of emergency response and health system development. Addressing VL requires sustained political commitment and donor alignment to move beyond short-term campaigns toward long-term integration within primary care. It also demands that regional and international partners recognise the disease as a proxy for equity and resilience rather than a narrow clinical problem. The goal should not only be reducing cases but building systems capable of detecting, treating, and preventing NTDs amid instability and climate stress.

As climate variability intensifies and pastoral mobility patterns shift, the ecological and epidemiological landscape of VL will likely expand. Failure to act now will entrench the disease as a marker of systemic inequity. Conversely, integrated action on VL offers a unique entry point for laying the foundations of universal health coverage in fragile settings—by linking community-based care, surveillance, and environmental management under a shared One Health umbrella. Eliminating VL in the Somali Peninsula is therefore not only a matter of disease control but a litmus test for political will and the global commitment to leave no one behind.
